# Cyber-Physical Geographical Information Service-Enabled Control of Diverse *In-Situ* Sensors

**DOI:** 10.3390/s150202565

**Published:** 2015-01-23

**Authors:** Nengcheng Chen, Changjiang Xiao, Fangling Pu, Xiaolei Wang, Chao Wang, Zhili Wang, Jianya Gong

**Affiliations:** 1 State Key Laboratory for Information Engineering in Surveying, Mapping and Remote Sensing, Wuhan University, Wuhan 430079, China; E-Mails: cjxiao@whu.edu.cn (C.X.); xiaolei8788@whu.edu.cn (X.W.); c.wang@whu.edu.cn (C.W.); gongjy@whu.edu.cn (J.G.); 2 School of Electronic Information, Wuhan University, Wuhan 430079, China; E-Mails: flpu@whu.edu.cn (F.P.); wangzhili1201@126.com (Z.W.); 3 Collaborative Innovation Center of Geospatial Technology, Wuhan 430079, China

**Keywords:** cyber-physical, geographical information service, sensor web, location-based instant sensing, open control, online control, closed-loop

## Abstract

Realization of open online control of diverse *in-situ* sensors is a challenge. This paper proposes a Cyber-Physical Geographical Information Service-enabled method for control of diverse *in-situ* sensors, based on location-based instant sensing of sensors, which provides closed-loop feedbacks. The method adopts the concepts and technologies of newly developed cyber-physical systems (CPSs) to combine control with sensing, communication, and computation, takes advantage of geographical information service such as services provided by the Tianditu which is a basic geographic information service platform in China and Sensor Web services to establish geo-sensor applications, and builds well-designed human-machine interfaces (HMIs) to support online and open interactions between human beings and physical sensors through cyberspace. The method was tested with experiments carried out in two geographically distributed scientific experimental fields, Baoxie Sensor Web Experimental Field in Wuhan city and Yemaomian Landslide Monitoring Station in Three Gorges, with three typical sensors chosen as representatives using the prototype system Geospatial Sensor Web Common Service Platform. The results show that the proposed method is an open, online, closed-loop means of control.

## 1. Introduction

Rajkunmar *et al.* define cyber-physical systems (CPSs) as physical and engineered systems the operations of which are monitored, coordinated, controlled and integrated using a computing and communication core. They assert that any systems that bridge the cyber-world of computing and communication with the physical world can be referred to as CPSs. They believe that just as the Internet transformed how human beings interact and communicate with one another, CPSs will transform how human beings interact with and control the physical world around us [1–3]. However, CPS research is still in its infancy. The U.S. National Academy of Engineering has listed 14 grand challenges that relate to environmental, health, and societal issues which will clearly benefit from advances achieved in CPSs [4].

CPSs pay much attention to control of sensors in the physical world that are networked and/or distributed, with feedback loops [5,6] in which sensing behaviors affect computations and *vice versa* [7,8], to bring the output of the sensors back to original or desired response, which can be referred to as closed-loop control [9]. However, according to analyses in Section 2, current methods of control, when applied to sensors, have the following drawbacks that hinder the development of CPSs:

(1) Current control methods lack openness. Openness is a measure of the extent to which a system comprises components that are built to Open Standards (e.g., OGC's OpenGIS Specifications). Current control methods have basically been developed for, and are applicable to, closed systems, such as Wireless Sensor Networks (WSNs). They usually provide direct communications between controllers and controlled devices in a local area network, through which the commands flow to target devices, instead of incorporating open standards to realize the corresponding functions. Thus control methods are applicable only to proprietary-control applications using specific communication protocols, which reduces or even eliminates reusability and interoperability of the control functions of target devices.
(2) Current control methods provide weak online and distributed control. “Online” here means on the Web which is a global area network. “Distributed” here refers to a control mode that all geographically distributed sensors are controlled by one master node through the Web at any place, which is different from traditional definition [10]. Most intra-buses in current industrial control application networks are based on industrial control buses and independent subsystems within the networks have difficulties connecting with each other through open buses or the Web, at a limited communication range, and with relatively weak communication capabilities. These weaknesses limit control operations to a relatively fixed and small area. Therefore, current control methods do not well support control of geographically distributed sensors through the Web. This results in inconvenient control.

The Sensor Web, first proposed by Delin and Jackson [11], as a smart macro instrument for coordinated sensing [12], consists of sensor nodes that not only collect data, but also share data and adjust their behaviors based on shared data. It enables an interoperable usage of sensor resources, hiding the underlying layers, the communication details, and heterogeneous sensor hardware, from applications built on top of it [13,14], through information models including Sensor Model Language (SensorML) [15,16] for describing sensor resources and Observations & Measurements Schema (O&M) [17–19] for describing sensor observations, and service interface specifications including Sensor Observation Service (SOS) [20–22] and Sensor Planning Service (SPS) [23–25], leveraging the models and encodings to allow accessing sensor data, tasking and control of sensors connected to the web [13,26–31]. Thereby, the models and services of Sensor Web can be adopted as middleware and incorporated into CPSs to provide open, interoperable control based on location-based instant sensing.

Sensor Web can provide geographical information service in a broad sense, just as other geographical information public service platforms such as Google Earth in the USA and Tianditu [32,33] developed by National Administration of Surveying, Mapping and Geoinformation of China (NASG) do. These geographical information services combine with CPSs to form cyber-physical geographical information service, providing the ability to manipulate geo-referenced sensors in the physical world through cyberspace and geographical information technologies based on location-based instant sensing of physical sensors according to predefined rules in an open way.

To solve the aforementioned problems related to sensor control and achieve open and online distributed control of diverse *in-situ* sensors, this paper proposes a cyber-physical geographical information service-enabled method for controlling diverse *in-situ* sensors that are connected to the Web, based on location-based instant sensing of these sensors. The method adopts the concepts and technologies of CPSs to combine control with sensing, communication, and computation, utilizes Web services and geographical information services, including Sensor Web and Tianditu as middleware, and builds well-designed HMIs to support online and open interactions between human beings and physical sensors. The method works due to the openness, interoperability, and reusability of Sensor Web services and Web services, distributed and open control features of CPS, and excellent geo-referenced visualization of target devices from Tianditu. The study offers the following contributions:

(1) An open and interoperable control architecture enabled by cyber-physical geographical information service. The architecture comprises a physical part and a cyber part. The physical part consists of diverse *in-situ* sensors. The cyber part includes five layers: a middleware layer, a Web services layer, a Sensor Web services layer, a Geographical Information Service-enabled applications layer and the HMI. The architecture contributes to open, online, distributed, and closed-loop feedback-based control.
(2) A self-adaptive thread sleep-wake (S-ATS-W) algorithm and an adjust thread sleep time (ATST) algorithm. These two algorithms work in union, responding quickly to commands to change the frequency of sensor data transmissions. Combining the two algorithms with geographical information service (e.g., Tianditu), geo-control of geo-referenced sensors within chosen spatial extent of interest is supported, which is a highlight over traditional sleep-wake scheduling algorithms in WSNs.
(3) Experiments performed in two scientific experimental fields: Baoxie Sensor Web Experimental Field in Wuhan city and Yemaomian Landslide Monitoring Station in Three Gorges. We conduct experiments in two stages: location-based instant sensing and open closed-loop control, during which we successfully tested the proposed method in real world scenarios.

The remainder of the paper is organized as follows: in Section 2, we review related work on the control of sensors. In Section 3, we provide the design of our proposed cyber-physical geographical information service-enabled control method for diverse *in-situ* sensors, and in Section 4 we describe how to implement the method. In Section 5, we present an experiment to test the proposed method. In Section 6, we discuss the advantages and limitations of the proposed method. And in Section 7, we provide conclusions with directions of future work.

## 2. Related Work

In recent years, much work has been done on control of sensors, and many control systems have been developed in real-world applications. A volcano-monitoring interdisciplinary project performed by the Harvard Sensor Network Lab is investigating the use of wireless sensor networks for monitoring eruptions of active and hazardous volcanoes. Researchers at the lab have deployed three wireless sensor networks on active volcanoes, which capture continuous seismic and acoustic signal data. A long-distance radio link between the observatory and the sensor networks is established to let their laptops monitor and control the network's activity, and a Java-based graphical user interface (GUI) is developed to monitor the networks' behavior and manually set parameters, such as sampling rates and event-detection thresholds [34,35]. Gutiérrez *et al.* [36] designed a Smart House network for its integration into a sustainable and bioclimatic solar house. It focused on a specific aspect of the house design, the control system bus, developed for the management of the different parameters, variables, sensors and actuators which coexist at home. A user interface was designed to manage the orders given by the user to the house and monitors the status of the system. Palma *et al.* [37] presented a way in which classroom control is accessed through Near Field Communication (NFC) and the information is shared via radio frequency. It develops an application that collects information from the classroom to create a control classroom tool that displays access to and the status of all the classrooms graphically and also connects this data with social networks. Mohamaddoust *et al.* [38] designed a Lighting Automatic Control System (LACS), which contains a centralized or distributed architecture determined by application requirements and space usage. The system optimizes the calculations and communications for lighting intensity, incorporates user illumination requirements according to their activities and performs adjustments based on external lighting effects in external sensor and external sensor-less architectures. Hwang *et al.* [39] realized a ubiquitous hog farm system that applies wireless sensor network technology to the pig industry to solve problems such as high mortality rates, increase productivity, and produce high quality pork. They suggest that a WSN and closed-circuit television (CCTV) should be installed on hog farms to collect environmental and image information that will help producers not only monitor the hog farm via the Web from outside the farm, but also control hog-farm facilities from remote locations. The system also allows facilities to be automatically controlled based on breeding environment parameters which are already set up and a short message service (SMS) notice service to conveniently notify users of deviations. The system consists of three layers: a physical layer, a middle layer, and an application layer. Park *et al.* [40] studied an automatic control system for greenhouse based on WSN to increase the productivity and reduce the chance of crop disease. They develop a system that consists of sensor nodes for temperature, humidity, leaf temperature, and leaf humidity, as well as a database server for storing collected data and relay nodes that use environmental information collected in real time to automatically control equipment such as windows, heaters, and ventilators.

These researches are nice work in realizing control of diverse sensors in small area of network to better utilize these sensors for specific purposes, for example, monitoring. However, these control methods are limited to WSN, which can hardly be used online (through the Web) at any place with any devices. Besides, they are not open enough and do not well support interoperable control, and many of them do not support closed-loop and distributed control, as is summarized in Table 1. These limitations lead to inconvenient control and reduce reusability and interoperability of control function.

## 3. Architecture Design of the Proposed Control Method

**Figure 1.**
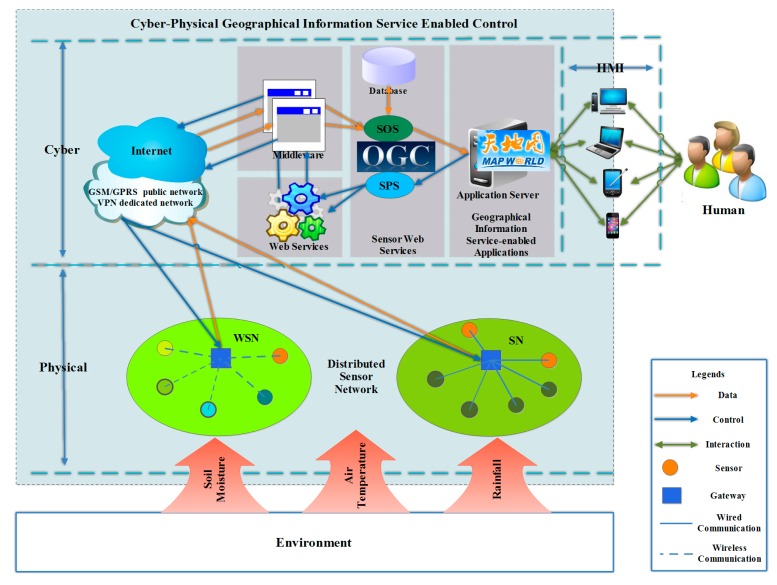
Architecture of cyber-physical geographical information service-enabled control of diverse *in-situ* sensors.

The proposed cyber-physical geographical information service-enabled control method uses CPS, and thus can be divided into two main parts, a physical part and a cyber part, as depicted in Figure 1. The cyber part comprises five components. To put this in another way, the cyber part is a five-layered architecture. The five layers are the middleware layer, Web services layer, Sensor Web services layer, Geographical Information Service-enabled applications layer, and the HMI. The proposed architecture can relate to the 3-tier Sensor Web layer stack introduced by Bröring *et al.* in [13], which is widely accepted, including sensor layer, Sensor Web layer, and application layer. A mapping can be established between the separation into physical part and cyber part as introduced here in the cyber-physical geographical information service-enabled control architecture, and the established layers in the 3-tier Sensor Web layer stack: (1) the physical part can be mapped to sensor layer; (2) the cyber part can be mapped to the Sensor Web layer and application layer. To be more specific, the middleware layer, Web service layer, and Sensor Web service layer can be mapped to the Sensor Web layer, and the Geographical Information Service-enabled application layer and the HMI can be mapped to application layer.

The physical part mainly consists of diverse *in-situ* sensors in distributed sensor networks, sensing the physical world, namely, measuring various environmental factors. The cyber part comprises many kinds of information technologies, and hides the physical world from users, making it virtual and transparent to users. The physical and cyber parts communicate with each other using wireless and wired communication, such as Global System for Mobile Communications (GSM) and general packet radio service (GPRS) public networks, virtual private networks (VPNs) over GPRS, and the Internet.

Sensor data from the physical part is transmitted to the cyber part and parsed, encoded by middleware, published to SOS, and eventually used by various geographical information service-enabled applications for chart display or high-level analysis applications such as instant thematic mapping. These applications interact with users through well-defined and well-designed HMIs, providing means for users to manipulate the physical part through the information world of the cyber part, based on computing results and decision rules. The manipulation command flows through geographical information service-enabled applications to SPS, invoking Web services based on the appropriate logic, and then flows to middleware before ultimately arriving at the physical part and adjusting behaviors of sensors. Different components in the cyber part communicate with each other mainly through the Internet, using Internet-related protocols.

### 3.1. Physical Part

The physical part is composed of various kinds of sensors, with different interface standards and communication protocols, and for different monitoring purposes. For example, barometers are used to monitor atmospheric pressure, rain gauges measure rainfall, three-dimensional electronic compasses monitor landfill, and particle sensors acquire particulate matter 2.5 (PM_2.5_) concentration. Sensors can be in distributed sensor networks (SNs) deployed in different areas, with fairly different environmental conditions, as long as data sensed by them can be transmitted back through communication channels (e.g., 3G or GPRS).

### 3.2. Cyber Part

#### 3.2.1. Middleware Layer

The middleware layer consists of diverse middleware programs that bridge the physical part and Sensor Web service layer, as well as the Web service layer. The middleware can be in different forms, ranging from desktop programs to windows service programs, and deployed in a distributed way, namely on different network hosts. The middleware has two main tasks: (1) acquire observations from diverse *in-situ* sensors in the physical part, encodes it in a standard encoding format (in our proposed method, we use O&M), and publishes the encoded observation to SOS; (2) get commands from SPS through Web services in the Web service layer and translate them to what can be understood by sensor controllers, and then make them executed.

#### 3.2.2. Web Service Layer

The Web services layer comprises various kinds of Web services that communicate with the middleware programs in the middleware layer and the SPS service in the Sensor Web service layer, namely, all control commands transmitting from SPS to middleware programs through web services. The Web services also allow users to query about the current observational behaviors of sensors. They are often deployed on the same network host as the middleware programs they work with.

#### 3.2.3. Sensor Web Service Layer

The Sensor Web service layer contains standard and open Sensor Web services developed by OGC SWE, especially SOS and SPS, with specifications implemented suitable for discovery, exchange, and processing of sensor observations, as well as the tasking and control of sensors.

SOS in this layer obtains observations sent from middleware layer and stores them in a background database that is transparent to users. SOS can be deployed on one host or more than one in a distributed way. When SOS is deployed on one host, all middleware programs in the middleware layer send encoded observations to one target SOS. When SOS is deployed on multiple hosts, each middleware program is bound with its own target SOS that may be different from others'. The former kind of deployment is often the case.

SPS in this layer plays a vitally important role in interoperable control of sensors or sensor systems through different suitable scheduling and control algorithms that call Web services in the Web service layer remotely and the control eventually takes effect through middleware programs in middleware layer.

#### 3.2.4. Geographical Information Service-Enabled Application Layer

The Geographical Information Service-enabled application layer is composed of various applications enabled by geographical information service running on different platforms, including desktop computers, laptops, PDAs, and smart cell phones, *etc*. The Geographical Information Service-enabled applications monitor environmental factors and control data collection/transmission frequencies of sensors in chosen spatial extent of interest.

Applications in this layer—the ultimate consumers of sensor data—deal with services exposed by the Sensor Web service layer. The applications request observations from SOS. They extract observation result values, geo-positions, observation times, *etc.* from SOS responses and use them in appropriate ways, such as displaying them in dynamic chart views to show instant status of the observed object, producing instant thematic maps to obtain environmental conditions in a given area; or making and submitting task plans to control the data acquisition process of diverse *in-situ* sensors in an interoperable way.

#### 3.2.5. HMI

HMIs provide interfaces for users to interact with sensors in the physical world through cyber space. HMIs can display instant observations from the physical world and provide control panels for users to manipulate sensor behaviors in the physical world according to obtained information and system rules.

## 4. Implementation of the Proposed Control Method

The proposed control method is based on location-based instant sensing of diverse *in-situ* sensors. Users take specific control strategies based on observations of sensors, as well as requirements and rules. Thereby, the proposed control can be regarded as closed-loop control, comprising two stages: location-based instant sensing, and open closed-loop control.

### 4.1. Location-Based Instant Sensing

**Figure 2.**
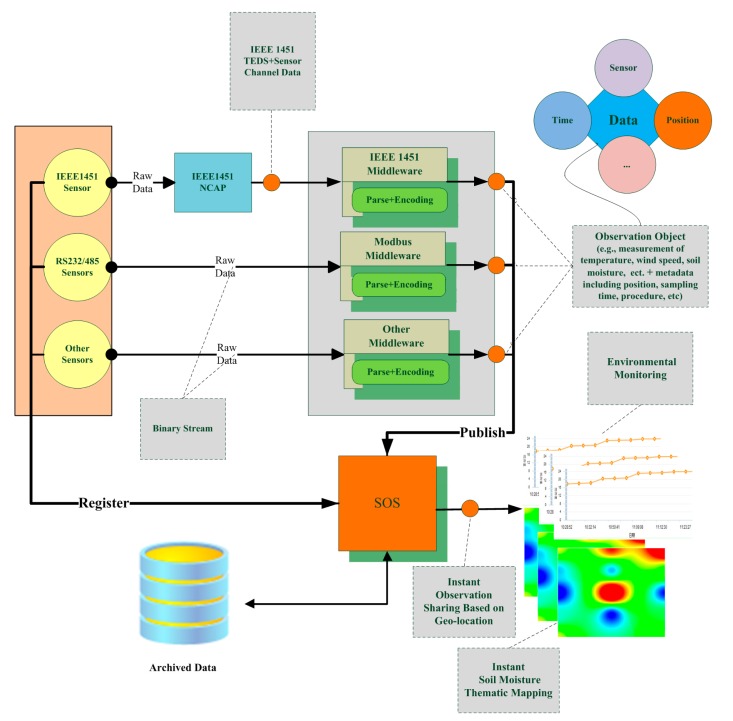
Location-based instant sensing of diverse *in-situ* sensors.

Location-based instant sensing is a process of acquiring and publishing sensor observations that carry location information instantly. As with our method, sensors must complete four steps to publish and share their originally sensed data: register in SOS, parse and encode instant observation data, publish the encoded data to SOS, and ultimately share their data instantly and serve user applications (see Figure 2). In detail, the steps are as follows:

(1) Registration of the sensor in SOS. Sensor observation can only be inserted for sensors that have been registered with SOS. For a sensor to be registered, a RegisterSensor request, including a sensor system description, must be sent. A sensor system description might be a SensorML document, and an O&M Observation instance that is a template for the observations that will be published for this sensor.
(2) Parsing and encoding of instant observation data. Original sensor data is often stored in storage modules of sensor systems, such as registers, in binary format. Middleware programs first have to send data acquisition requests to sensors or sensor systems in a predefined format periodically. When response data returns, validation should be performed with appropriate methods to filter out incorrect data. A cyclic redundancy check (CRC) is a commonly used error-checking method. If data is valid, it is parsed according to the predefined format and encoded in O&M format, with metadata such as observation location and time added. If data is invalid, it is discarded and the next data request is sent immediately to avoid meaningless waiting.
(3) Publishing of encoded data to SOS. After observation data has been encoded, it is inserted into SOS using the InsertObservation operation through the POST method in a Hyper Text Transfer Protocol (HTTP) web request.
(4) Sharing of sensor data instantly and serving user applications. When data has been published to SOS, it can serve various user applications through standard service interfaces in various forms, including direct display in charts, overlapping on top of navigable maps, and other forms of further high-level analysis.

### 4.2. Open Closed-Loop Control

Open control is essentially interoperable control—here realized by making use of SPS open standard service interfaces. Closed-loop control is a control decision process based on instant sensing of sensors, together with certain requirements and rules.

**Figure 3.**
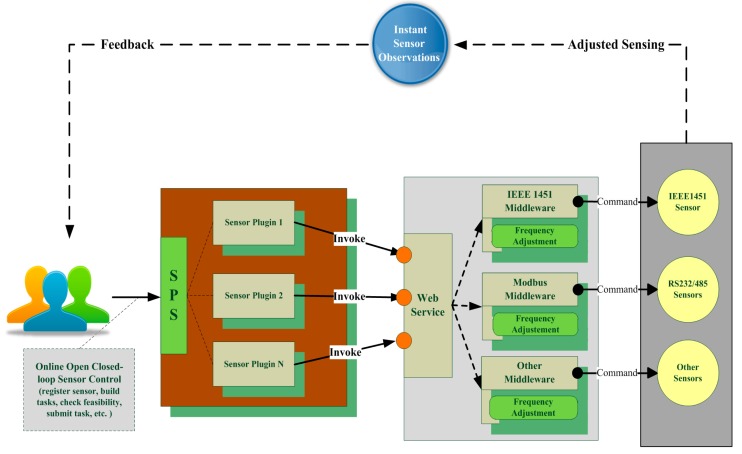
Open closed-loop control for diverse *in-situ* sensors.

Open closed-loop control is achieved through four steps: (1) installing the plugin and registering sensor; (2) making and submitting control plans according to instant sensor observations; (3) invoking appropriate Web services and controlling real sensors through middleware programs; (4) real sensors acting in response to commands, as depicted in Figure 3. The steps are described in detail in the following subsections.

#### 4.2.1. Installing Plugin and Registering Sensor

First, multiple sensor plugins should be developed for each sensor type or sensor platform type and installed in SPS. Once integrated into the SPS framework, these plugins with concrete control strategies can provide the SPS interface for a certain type of sensor or sensor platform. Then, a sensor instance, belonging to a certain kind of sensor plugin installed, must be registered in SPS.

Sensor registration information includes the plugin type a sensor instance belongs to, an alias for the instance, configuration information, and input parameter descriptions of the instance, being registered using the Register request.

#### 4.2.2. Making and Submitting Control Plans according to Feedbacks

When a sensor instance has been registered, control plans can be made according to input parameter descriptions in the response of a DescribeTasking request. On the other hand, control plans are made according to feedback from instant sensor observations or high-level analysis results based on those observations, and certain requirements in that circumstances, together with decision rules. Then feasibility of the control request must be checked using the GetFeasibility operation. If feasible, the control request can be submitted using the Submit operation. Otherwise, users have to reconstruct the control request and check its feasibility until it is feasible.

#### 4.2.3. Invoking Appropriate Web Services and Controlling Real Sensors through Middleware

For each sensor plugin installed in the SPS framework, a control strategy is included. The strategy includes logic for invoking appropriate Web services, which allows communication with middleware that has direct contact with the target sensor or sensor platform. These Web services are designed for a certain kind of sensor or sensor platform each. Web services that get/set sensor data collection/transmission frequencies are often the case.

Whether a sensor or sensor platform has a module for controlling the frequency with which data is collected or not affects how Web services interact with middleware programs and, ultimately, the sensor or sensor platform.

- Sensors and sensor platforms without modules for controlling the frequency with which data is collected: In this case, the SPS plugin first calls Web services in appropriate logic to query about the current data transmission frequency that is stored in the database (table design is shown in Table 2, with two fields “SENSOR_ID” and “INTERVAL” whose data types are text and double precision number respectively) deployed on a network host. If the current transmission frequency is the same as what is going to be set, no further action will be taken. Otherwise, the new data transmission frequency will be updated for the specified sensor with the given SENSOR_ID value. Meanwhile, the middleware program queries the database at a preset frequency in a configuration file and adjusts the data transmission frequency with delay just around the time of queryDBInterval (an input of Algorithm 2) dynamically based on Algorithm 1, working in union with Algorithm 2. As the middleware program rapidly responds to commands to change the data transmission frequency, commands take effect on the real sensors quickly. As is often the case, the above process can be finished in quite a short period of time, but due to network congestion and limitations of network bandwidth sometimes, it can take a long time to react to the command to change the frequency of sensor data transmission frequencies.
- Sensors and sensor platforms with modules for controlling the frequency with which data is collected: In this case, the SPS plugin calls Web services in appropriate logic to query about the current data collection frequency that is stored in a database (Table designs are shown in Tables 3 and 4. Table 3 has six fields: “ORDER_NUMBER”, “SENSOR_ID”, “ORDER”, “SAMPLING”, “LOOP”, and “ORDER_RECEIVE_DATE”, representing the number of inserted order, sensor identification, order type, data collection interval, collection times in a cycle, and the date and time when the order was received, respectively. Table 4 has three fields: “ORDER_NUMBER”, “EXECUTE”, and “TIME”, representing the number of inserted order, whether the order was executed successfully, and the time when the execution status of the order was changed, respectively) deployed on a network host. If the current collection frequency is the same as what is going to be set, no further action is taken. Otherwise, a new order is inserted to the order table (Table 3). At the same time, the middleware program queries the order table for the latest order and sends it to the Microprogrammed Control Unit (MCU), which translates the order to what can be understood by the sensor. The sensor then acts on the order, changing its collection status. Whether the order is executed successfully or not, an order execution status record is inserted into Table 4.

**Algorithm 1** Self-adaptive thread sleep-wake (S-ATS-W) algorithm, which is used to rapidly responds to commands to change the frequency with which sensor data is transmitted
**Input:** database connection string connectionString sensor identification sensorid**Output:** void**Use:** GetSqlConnection(connectionString) to get a database connection object using the given connection string  CloseSqlConnection(conn) to close the database connection that connection object conn holds to release database resource  GetTransIntervalBySensorID(conn, sensorID) to get sensor data transmission interval from database  GetQueryDBIntervalFromCfg() to get database query interval from external configuration file  ATST(transInterval, queryDBInterval, sleptTime, connectionString, sensorid) algorithm defined thereafter to dynamically adjust the length of sleep time of a thread**Declare:** SqlConnectionObject conn**Begin:**1: conn = GetSqlConnection(connectionString)2: **If** (conn! = null) **then**3:{double transInterval = GetTransIntervalBySensorID (conn, sensorid); CloseSqlConnection(conn);4: double queryDBInterval = GetQueryDBIntervalFromCfg();5: ATST(transInterval, queryDBInterval, 0, connectionString, sensorid)}6: **End if****End**


**Algorithm 2** Adjust thread sleep time (ATST) algorithm, which is used to dynamically change the length of sleep time of a thread
**Input:** current data transmission interval transInterval, which is a double precision number time interval that the database storing sensor data transmission frequencies is queriedqueryDBInterval, which is double precision number total time a thread has slept for sleptTime, which is a double precision number database connection string connectionString sensor identification sensorid**Output:** void**Use:** GetSqlConnection(connectionString) to get a database connection object using the given connection string  CloseSqlConnection(conn) to close the database connection that connection object conn holds to release database resource  GetTransIntervalBySensorID(conn, sensorID) to get sensor data transmission interval from database  GetQueryDBIntervalFromCfg() to get database query interval from external configuration file  ThreadSleep(timeInterval) to make a thread sleep for time timeInterval**Declare:** None**Begin:**1:**If** (indicationInterval <= queryDBInterval) **then**2:{ThreadSleep(transInterval);}3:**Else**4:{ThreadSleep(queryDBInterval);5: conn = GetSqlConnection(connectionString);6: double newTransInterval = 0.0;7: **If** (conn! = null) **then**8: {newTransInterval= GetTransIntervalBySensorID(conn, sensorid);9: CloseSqlConnection(conn);}10: **End If**11: sleptTime = sleptTime + queryDBInterval;12: **If** (newTransInterval > sleptTime) **then**13: {newTransInterval = newTransInterval- sleptTime;14: queryDBInterval = GetQueryDBIntervalFromCfg();15: ATST(newTransInterval, queryDBInterval, sleptTime, connectionString, sensorid);}16: **End If**}17:**End If****End**


#### 4.2.4. Real Sensors Acting in Response to Commands

Once real sensors receive commands, they act on the commands to adjust their data collection frequencies or transmission frequencies. For sensors without data collection frequency control modules, their data collection frequencies are usually fixed before delivery. On the other hand, the predominant power consumption is in the process of communication—namely data transmission [41–44]. Therefore, controlling the data transmission frequency is the main way to save power. A sensor collects environmental data on its routine, and when a data request arrives, it retrieves data from registers according to requirements and sends the data to the requester immediately. This process repeats periodically according to the data transmission frequency preset in the middleware program configuration.

For sensors with data collection frequency control module, their data collection frequencies can be changed easily at any time using the MCU, which gets latest order pushed by the middleware program hosting on a remote computer (also referred to as an upper computer or principle computer) through a communication link. If the sensor receives a “sleep” command, it stops data collection until a “wake up” command arrives; otherwise, it collects data at the newly given frequency and sends data back to the middleware program through the established communication link in the predefined format.

## 5. Experiments

In order to test practicability and suitability of the proposed method in real world applications, we performed an experiment at two geographically distributed scientific experimental fields far from each other. The experiment consisted of two stages: (1) location-based instant sensing of diverse *in-situ* sensors; (2) control of sensors based on the collected data. More details regarding the experiment are provided in the following subsections.

### 5.1. Experiment Scenarios

#### 5.1.1. Overview of Two Scientific Experimental Fields

**Figure 4.**
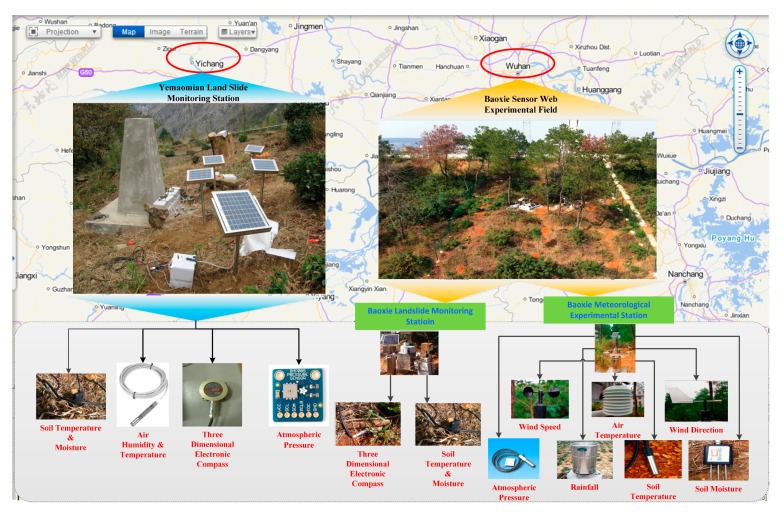
Two scientific experimental fields.

To perform the experiment, we chose two scientific experimental fields in China: Baoxie Sensor Web Experimental Field and Yemaomian Landslide Monitoring Station in Hubei Province. The first field is in the town of Baoxie (30.47023° N, 114.52685° E), Wuhan, and the second is in Yemaomian (30.89306° N, 110.86667° E), Three Gorges. An overview of these two scientific experimental fields is shown in Figure 4.

The Baoxie Sensor Web Experimental Field (the right experiment field in Figure 4) is mainly used for Sensor Web researches. It is about 800 m^2^ in area, with 70 sensors. There are 14 types of sensors: anemometer, barometer, thermometer, hygrometer, rain gauge, three-dimensional electronic compass, PH meter and so on. Environmental factors monitored include wind speed and direction, atmospheric pressure, air temperature and humidity, soil temperature and moisture, rainfall, angle of heading and pitch and roll, and so on. These sensors belong to four stations: Baoxie Landslide Monitoring Station, Baoxie Meteorological Experimental Station, Baoxie Edaphic and Meteorological Monitoring Station, and Baoxie Soil Temperature and Moisture Monitoring Station. As sensors belonging to the same station are not far away from each other, only the stations have Global Positioning System (GPS) module attached and the position of each station approximately represents the position of sensors connected to it. The sensors connect to the MCUs of the corresponding stations through wired connections that use GPRS to communicate with middleware programs deployed on a network host in the State Key Laboratory for Information Engineering in Surveying, Mapping and Remote Sensing (LIESMARS) at Wuhan University. All of the sensors are powered by photovoltaic solar energy.

The Yemaomian Landslide Monitoring Station (the left experimental field in Figure 4) is mainly used for landslide monitoring in Yemaomian, which is near the Three Gorges Reservoir. It is about 20 m^2^ in area, with five sensor nodes of four types: thermometer, hygrometer, barometer, and three-dimensional electronic compass. Environmental factors monitored include soil temperature and moisture, air temperature and humidity, and angle of heading, pitch, and roll. Each sensor node has a MCU and a GPS module. Sensors in a node connect to the MCU through wired connections. The sensor nodes communicate with a gateway node using ZigBee, which forms a WSN, while the gateway node uses 3G to communicate with middleware programs deployed on a network host in the School of Electronic Information (SEI) at Wuhan University. All of these sensors are powered by photovoltaic energy.

All of the sensors in both experimental fields transmit observations through multi-hop networks to a data center—SOS deployed on a server in LIESMARS ultimately.

#### 5.1.2. Sensors Selection

**Figure 5.**
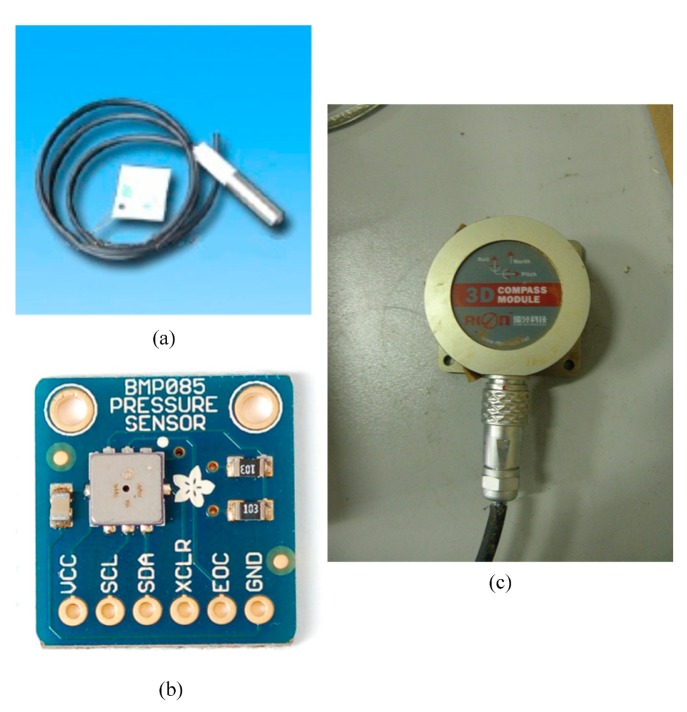
(**a**) Non-IEEE1451-based Barometer in Baoxie Sensor Web Experimental Field; (**b**) IEEE1451-based barometer at Yemaomian Landslide Monitoring Station; (**c**) IEEE1451-based three-dimensional electronic compass at Yemaomian Landslide Monitoring Station.

In order to carry out the experiment convincingly, sensors selection is of vital importance. Here, we chose two barometers and a three-dimensional electronic compass. One of the two barometers was an IEEE1451-based smart sensor deployed at Yemaomian Landslide Monitoring Station, while the other was a non-IEEE1451-based common sensor (not smart sensor) deployed in Baoxie Sensor Web Experimental Field. The three-dimensional electronic compass that we selected was an IEEE1451 based smart sensor deployed in Yemaomian Landslide Monitoring Station. These sensors are shown in Figure 5 and the details of their specifications are listed in Table 5.

We chose the above three sensors based on four considerations: (1) We wanted sensors built to diverse interface standards (a barometer built according to the IEEE 1451 standard and one not built to the standard) but monitoring the same environmental factor (atmospheric pressure); (2) We wanted multiple sensors built to the same interface standard but monitoring different environmental factors, *i.e.*, an IEEE1451-based barometer and an IEEE 1451-based three-dimensional electronic compass to monitor atmospheric pressure and landslides respectively; (3) We wanted one sensor that monitors diverse environmental factors, *i.e.*, a three-dimensional electronic compass that monitors the angle of heading, pitch, and roll; (4) We wanted sensors that are deployed in distributed areas far from each other and under different environmental conditions, *i.e.*, one sensor in Baoxie Sensor Web Experimental Field and two sensors in Yemaomian Landslide Monitoring Station.

#### 5.1.3. Prototype System

We developed the prototype system for a web environment. For the three chosen sensors, we developed two middleware programs: one for the LYQYZ31 barometer and one for the BMP085 barometer and DCM308 three-dimensional electronic compass. We adopted SOS1.0 and SPS1.0 developed by 52North as the implementation frameworks and bases for further development. We developed a cyber-physical geographical information service application, Geospatial Sensor Web Common Service Platform, to facilitate interactions with deployed geo-referenced sensors, display of sensor data, and control of sensors. It is a mash-up application that combines sensor streams obtained using Sensor Web with maps from Tianditu of NASG. Details of the implementation of the prototype system are provided in Table 6.

### 5.2. Experiment Processes and Results

As was stated at the beginning of this section, the experiment comprised two phases: (1) location-based instant sensing; (2) closed-loop control based on sensed data.

#### 5.2.1. Location-Based Instant Sensing

Though three sensors were chosen to perform the experiment, here we use the LYQYZ31 barometer to illustrate the process from sensing to publishing and sharing of sensed data. The process is almost identical for the other two sensors.

**Figure 6.**
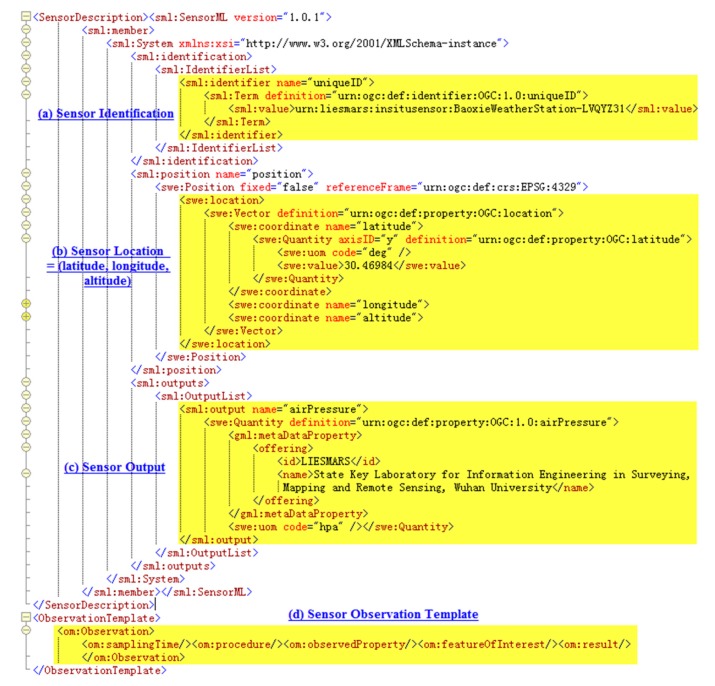
Description of LYQYZ31 barometer for registration.

**Figure 7.**
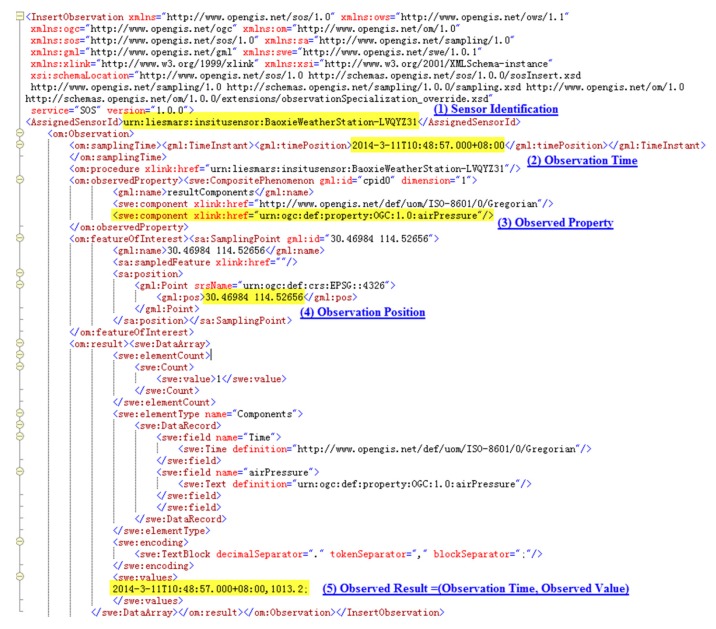
InsertObservation request for the LYQYZ31 barometer.

As in the steps described in Section 4.1, the LYQYZ31 barometer is first registered in SOS with the necessary metadata shown in Figure 6. Then a data request is sent to the MCU to which the barometer is attached by the middleware program using TCP/IP protocol through GPRS communication. When data returns, the middleware program converts it from binary format to a format that can be understood by human beings according the predefined data exchange format, encodes it into O&M format with metadata, and ultimately encapsulates it in an InsertObservation request, as shown in Figure 7. Here in the InsertObservation request, data acquisition time and location metadata are added as expressed by “<om:samplingTime><gml:TimeInstant><gml:TimePosition> 2014-3-11T10:48:57.000+08:00</gml:TimePosition></gml:TimeInstant><om:samplingTime>” and “<sa:position><gml:Point><gml:pos>30.46984 114.52656</gml:pos></gml:Point><sa:position>,” respectively.

**Figure 8.**
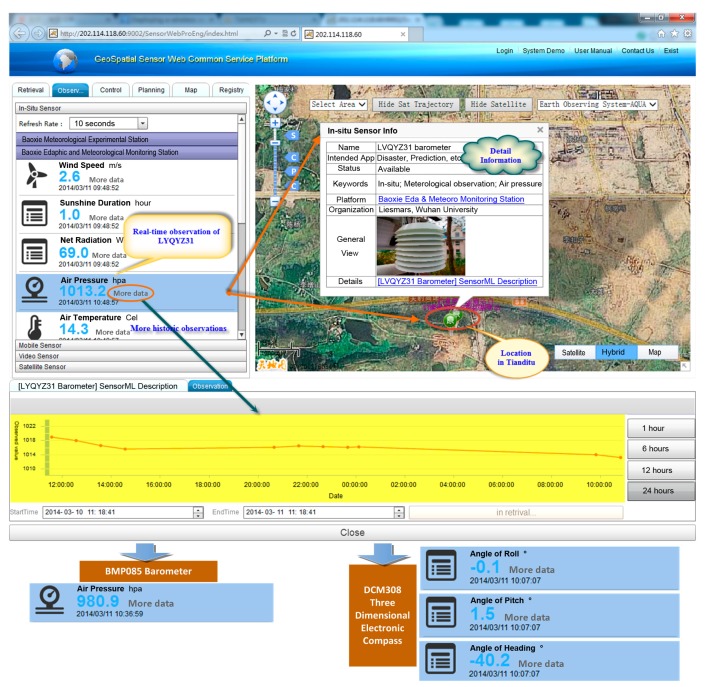
Geospatial Sensor Web Common Service Platform displaying location-based instant sensor observations.

Through all of the above steps, location-based instant sensor data is published to SOS. Then, the cyber-physical geographical information service application, Geospatial Sensor Web Common Service Platform, can acquire that data from SOS and display it in short delay, as is shown in Figure 8 (data from the other two sensors is shown at the bottom).

#### 5.2.2. Closed-Loop Control

In this phase, according to the steps described in Section 4.2, the SPS plugin for the LYQYZ31 barometer is first developed and installed in the SPS framework. Then a LYQYZ31 barometer instance is registered in SPS with the sensor configuration information, including plugin configuration, instance configuration, and input descriptions information.

**Figure 9.**
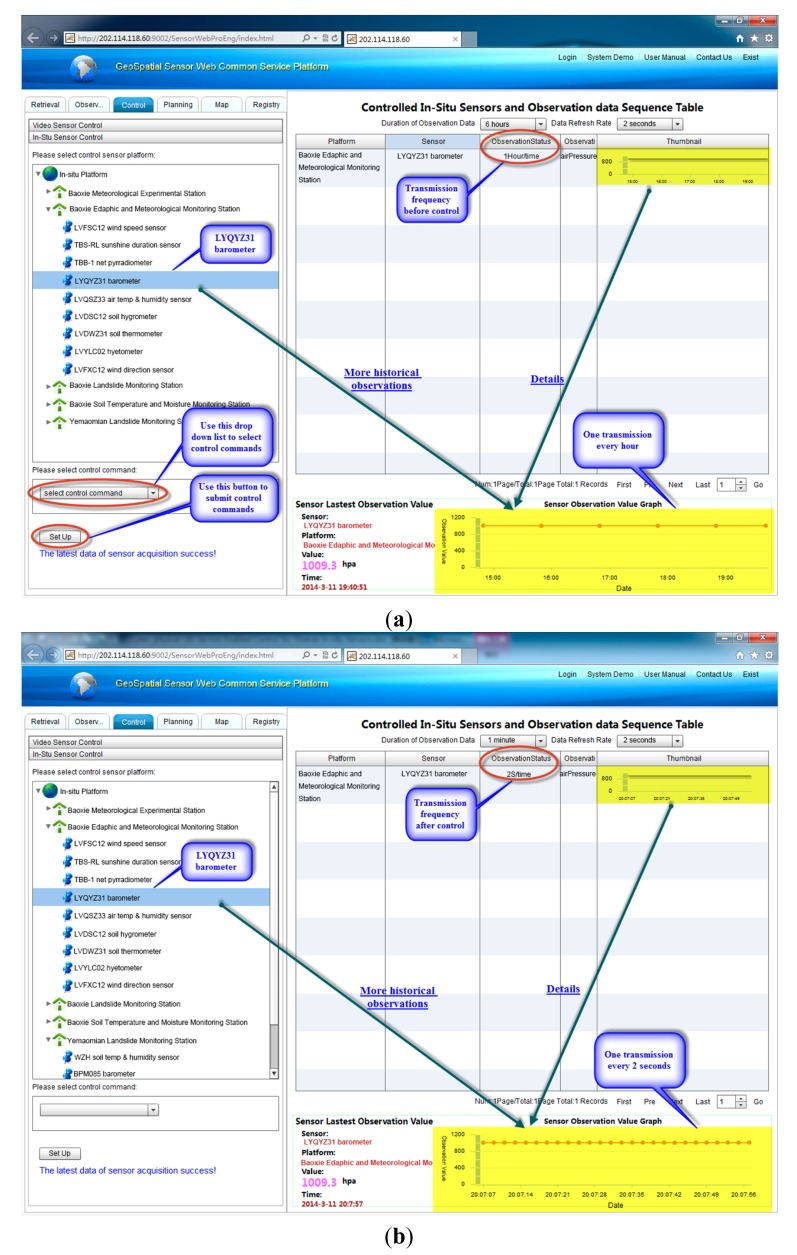
(**a**) Chart view of observations of the LYQYZ31 barometer before the frequency change; (**b**) Chart view of observations of the LYQYZ31 barometer after the frequency change.

Now assume that the transmission frequency for the LYQYZ31 barometer must be changed according to certain requirements. For example, consider that it must be changed from one transmission every hour to one transmission every two seconds to observe intensively, because acquired instant observation from LYQYZ31 is abnormal according to predefined standards and rules. To meet this requirement, a task to change the transmission frequency is set and the feasibility of the task is checked. Then the task is submitted to SPS and takes effect on the LYQYZ31 barometer. Figure 9a,b show the chart view of observations of the LYQYZ31 barometer before and after the frequency change respectively. The change of data collection frequency of LYQYZ31 is accomplished in less than 2 s.

This experiment, comprising two stages, *i.e.*, location-based instant sensing and closed-loop control based on sensed data, indicates that the proposed method is practical and suitable in real world applications, and can control geographically distributed diverse *in-situ* sensors with different communication protocols online through a web application.

## 6. Discussion

The proposed Cyber-Physical Geographical Information Service enabled control method of diverse *in-situ* sensors has the following advantages (detailed from Section 6.1 to 6.4) over other similar sensor control methods.

### 6.1. Open Control

In comparison with current control methods [34–40] which have low openness, the proposed control method of diverse *in-situ* sensors is fairly open from an application-oriented perspective, as it adopts CPS concepts and technologies that are inherently open, as well as Sensor Web standard services and Web services (in the proposed control method, all control functions are encapsulated into and published as Web services, which are fairly easy and convenient for reuse in various applications across different platforms) that are characteristically open, and interoperable. The proposed control process is independent of individual physical sensors, individual systems, and individual device vendors. The middleware layer, Web services layer, and Sensor Web services layer cooperate tightly, “virtualizing” the physical sensors, hiding heterogeneous communication means, commands format *etc.*, and making them transparent to user applications, which provides a standard, uniform and interoperable way for user applications to control sensors in the physical world through cyberspace.

### 6.2. Online and Distributed Control

Compared to control methods [36–38,40], the proposed method provides online control of diverse *in-situ* sensors. It makes use of CPS architecture that emphasizes the connection of sensors in the physical world to the Web. Sensors are not limited to a local area network, but spread throughout the global area network—the Web. Therefore, all sensors can be controlled online remotely, utilizing various kinds of networked devices, such as desktop computers, laptops, PDAs, smart phones and so on, at any time in any place. Because all sensors are connected to the Web, all control logic can be integrated into one application, which facilitates the control of geographically distributed sensors in a centralized way, making the control process convenient and is thus a great advantage over that in [36,37,40].

### 6.3. Closed-Loop Control

The proposed control method is not aimless control but based on instant observations from the physical sensors. Users check these observations, and then refer to certain standards, requirements or system rules to determine what control strategies to develop and put them into reality through the proposed control method. Target sensors act on these control commands to adjust their sensing behaviors and this adjustment reflects in the instant observations based on which users further determine what to do next in another cycle. The above process can be summarized to the following sequence: receive sensor feedbacks > make decisions > control sensors > receive sensor feedbacks > make decisions > control sensors… This sequence of activities forms a closed-loop control that takes situations and requirements at the control time into consideration, which is not supported in [34–37].

### 6.4. Geo-Control

The proposed method utilizes geographical information service to perform geo-location related operations. All sensors have their locations (precise coordinates of latitude and longitude) registered in SOS, and so do all observations. Users can specify spatial extents of interest (usually a bounding box) in a geographical information public service platform (e.g., Tianditu), and further obtain sensors within that chosen area(s) by cooperating with SOS. Then, these target geo-referenced sensors can be controlled through published Web services that encapsulate the proposed S-ATS-W and ATST algorithms. Thus, the proposed control method supports geo-control of geo-referenced sensors, which is usually not supported in traditional control and schedule of sleep-wake state of sensors in WSNs for energy conservation [45,46].

However, though the proposed architecture is interoperable from an application-oriented perspective by adopting CPS concepts and technologies, as well as Sensor Web standard services and Web services, the gap of interoperability between underlying sensors in the physical part and Sensor Web services in the cyber part still exist. Sensors are integrated with the Sensor Web by manually building proprietary bridges for each pair of SWE services implementation and sensor type, for example, the middleware layer to bridge sensors with SOS, the middleware layer and Web services layer to bridge sensors with SPS, and the manually implemented SPS plugins. This is somehow cumbersome and leads to extensive adaption efforts especially in large-scale sensor network deployments.

## 7. Conclusions and Outlook

To solve the two problems facing control of diverse *in-situ* sensors, namely, lacking openness and interoperability, and being weak online control, the paper proposes a cyber-physical geographical information service-enabled control method. The architecture of the method consists of two parts: a physical part and a cyber part. Further, the cyber part includes five layers: middleware layer, Web service layer, Sensor Web service layer, Geographical Information Service-enabled application layer and the HMI. The method leverages CPS to integrate sensors in the physical world with information technologies in cyberspace, and in particular, utilizes geographical information service to build geo-sensor applications and assist in the display of geographically related information and geo-control of geo-referenced sensors. To respond quickly to commands to change the frequency of sensors data collection/transmission, a self-adaptive thread sleep-wake (S-ATS-W) algorithm and an adjust thread sleep time (ATST) algorithm are developed and encapsulated as Web services for web-based invocation. The change of data transmission frequency can be accomplished in less than 2 s using these two algorithms in union. The proposed method is tested with an experiment performed in two geographically distributed experimental fields far away from each other: Baoxie Sensor Web Experimental Field and Yemaomian Landslide Monitoring Station. To carry out the experiment, three typical sensors are chosen as representatives from among 14 types of sensors and a total of nearly 80 sensors deployed in these two experimental fields, as described in Section 5. Experimental results show that the proposed method realizes open online closed-loop control, and is practical and suitable in real world scenarios. The method provides a new perspective for solving problems facing control of diverse *in-situ* sensors as described in Section 1.

The proposed method is implemented for control of sleeping/waking up status and data collection/transmission frequency of diverse *in-situ* sensors. However, it's not limited to control of these behaviors of mentioned sensors. Other behaviors of more kinds of sensors can be controlled, for example, observation orientation of cameras. In the future, we consider to test the practicability and suitability in control of mobile sensors with our proposed method, such as the control of flight path of Unmanned Aerial Vehicle (UAV) through a web application.

As is discussed in Section 6, the proposed architecture is not fully open and interoperable from a sensor-oriented perspective. In our future work, SIDs [47,48] can be introduced in the cyber part and PUCK-enabled instruments [49] can be used in the physical part to help close the gap of interoperability between underlying sensors and Sensor Web services, and minimize the efforts of integration of sensors in physical part with Sensor Web services in cyber part. Besides, the semantics technologies [50–52] can also be incorporated into our proposed method to provide improved accessing and controlling of sensors. Other important issues such as traffic routing between sensors and into the backbone network, security and protection against misbehaviors and malware, as well as against denial of service attacks can also be studied in the future.

## Figures and Tables

**Table 1.** Features of current control systems.

**System**	**Openness**	**Online**	**Closed-Loop**	**Distributed**
Harvard Volcano-monitoring Sensor Network [34]	Low	Yes	No	Yes
Smart House Network [36]	Low	No	No	No
Classrooms Access Control System [37]	Low	No	No	No
Lighting Automatic Control System (LACS) [38]	Low	No	Yes	Yes
Ubiquitous Hog Farm System [39]	Medium	Yes	Yes	Yes
Greenhouse Automatic Control System [40]	Medium	No	Yes	No

**Table 2.** Database table that stores sensor and sensor platform data transmission frequencies.

	**SENSOR_ID [PK] Text**	**INTERVAL Double Precision**
1	*sensor_id_1*	*i_1_*
2	*sensor_id_2*	*i_2_*
3	*sensor_id_3*	*i_3_*
…	…	…
*n*	*sensor_id_n*	*i_n_*

*i* (*i* = 1, 2, 3, …, *n*) is the number of a record which is continuous and increases by 1 automatically each time a new record is inserted. *i_r_* (*r* = 1, 2, 3, …, *n*) represents the data transmission frequency of the sensor with sensor ID “*sensor_id_r*.”

**Table 3.** Database table that stores sensor and sensor platform data collection orders.

**ORDER_NUMBER [PK] Integer**	**SENSOR_ID Text**	**ORDER Integer**	**SAMPLING Double Precision**	**LOOP Integer**	**ORDER_RECEIVE_DATE Datetime**
1	*sensor_id_1*	*o*_1_	*s*_1_	*l*_1_	*dt_1_*
2	*sensor_id_2*	*o*_2_	*s_2_*	*l*_2_	*dt_2_*
3	*sensor_id_3*	*o*_3_	*s*_3_	*l*_3_	*dt_3_*
…	…	..	…	…	…
*n*	*sensor_id_n*	*o_n_*	*s_n_*	*l_n_*	*dt_n_*

*i* (*i* = 1, 2, 3, …, *n*) is the number of an order which is continuous and increases by 1 automatically each time a new order is inserted. *sensor_id_i* (*i* =1, 2, 3, …, *n*) represents the sensor ID of *i*th order. (*o_i_*, *s_i_*, *l_i_*) ϵ {(0, 0, 0), (1, 12, 6), (1, 22, 3)} (*i* = 1, 2, 3, …, *n*). *dt_i_* (*i* = 1, 2, 3, …, *n*) is the date time of reception of *i*th order in the format “YYYY-MM-DD HH-MM-SS” (e.g., “2014-02-13 13:53:18”).

**Table 4.** Database table that stores execution statuses of sensor and sensor platform data collection orders.

**ORDER_NUMBER [PK] Integer**	**EXECUTE Text**	**TIME Datetime**
1	*e*_1_	*dt*_1_
2	*e*_2_	*dt*_2_
3	*e*_3_	*dt*_3_
…	…	…
*n*	*e_n_*	*dt_n_*

*i* (*i* = 1, 2, 3, …, *n*) is the number of an order which is continuous and increases by 1 automatically each time a new record is inserted. *e_i_* ε {‘Y’, ‘N’} (*i* = 1, 2, 3, …, *n*) represents whether the *i*th order has been successfully executed, where “Y” represents “YES” and “N” represents “NO.” *dt_i_* (*i* = 1, 2, 3, …, *n*) is the date and time when the execution status of *i*th order was changed, in the format “YYYY-MM-DD HH-MM-SS” (e.g., “2013-02-13 13:53:18”).

**Table 5.** Specifications of three experimental sensors.

**Hardware Type**	**Specifications**
IEEE1451 Barometer (BMP085)	Pressure sensing range: 300–1100 hPa
	Resolution: 0.03 hPa/0.25 m
	Operational temperature range: −40 to +85 °C
	Temperature accuracy: ±2 °C
	Supply voltage: 1.8–3.6 V (V_DDA_); 1.62–3.6 V (V_DDD_)
	Power: 5 μA at 1 sample/sec. in standard mode
	Noise: 0.06 hPa (0.5 m) in ultra-low power mode; 0.03 hPa (0.25 m) in ultra-high resolution mode

Non-IEEE1451 Barometer (LVQYZ31)	Physical size: Φ 17 × 110
	Pressure sensing range: 10–1100 hPa
	Resolution: 0.1 hPa
	Accuracy: 0.5 hPa
	Operational temperature range: −40–125 °C
	Supply voltage: 6.5–9 V (DC)
	Current: <= 0.1 mA
	Interfaces: 1P: +5 V; 2P:GND; 3P:RS485 A; 4P:RS485 B
	Communication interface: RS485
	Communication protocol: compatible with Modbus

IEEE1451Three-Dimensional Electronic Compass(DCM308)	Physical size: L50 × 49 × 15 mm
	Inclination measuring range: ±80°
	Accuracy:0.8°
	Operational temperature range: −40–85 °C
	Operating current: 40 mA
	Communication interfaces: RS232/485

**Table 6.** Implementation details of Geospatial Sensor Web Common Service Platform.

**Item**	**Hardware**	**Operation System**	**Database**	**Web Application Server**	**Development Language**	**Type**
**Module**						
BMP085 Middleware	CPU: Intel(R) Xeon(R)CPU E5620@ 2.40 GHz 2.39 GHz RAM: 15.9.0 GB	Windows Server 2003 Enterprise Edition Service Pack 2	MySQL 5.5.17	-	Java	Desktop Application
DCM308 Middleware						
LYQYZ31 Middleware	CPU: Intel(R) Xeon(R) CPU E5-2650 0@ 2.00 GHz 2.00 GHz RAM: 32.0 GB	Windows Server 2008 R2 Enterprise 64bit	PostgreSQL 9.2.1	-	C#.NET	Windows Service
Geospatial Sensor Web Common Service Platform			-	Tomcat 7.0.42	Java; JavaScript ActionScript 3.0	Java Web Application
SOS			PostgreSQL 9.2.1		Java	
SPS			eXist-db 1.2.4			
